# The Golgi-Associated PDZ Domain Protein Gopc/PIST Is Required for Synaptic Targeting of mGluR5

**DOI:** 10.1007/s12035-021-02504-9

**Published:** 2021-08-12

**Authors:** Malte Klüssendorf, Inseon Song, Lynn Schau, Fabio Morellini, Alexander Dityatev, Judith Koliwer, Hans-Jürgen Kreienkamp

**Affiliations:** 1 Institute for Human Genetics, University Medical Center Hamburg Eppendorf, Hamburg, Germany; 2 Molecular Neuroplasticity Group, German Center for Neurodegenerative Diseases (DZNE), 39120 Magdeburg, Germany; 3 Research Group Behavioral Biology, Center for Molecular Neurobiology, University Medical Center Hamburg Eppendorf, Hamburg, Germany; 4 Center for Behavioral Brain Sciences (CBBS), 39106 Magdeburg, Germany; 5 Medical Faculty, Otto-Von-Guericke University, 39120 Magdeburg, Germany

**Keywords:** Trans-Golgi network, Long-term depression, mGlu5, Neuroligin, PDZ domain

## Abstract

In neuronal cells, many membrane receptors interact via their intracellular, C-terminal tails with PSD-95/*discs large*/ZO-1 (PDZ) domain proteins. Some PDZ proteins act as scaffold proteins. In addition, there are a few PDZ proteins such as Gopc which bind to receptors during intracellular transport. Gopc is localized at the trans-Golgi network (TGN) and binds to a variety of receptors, many of which are eventually targeted to postsynaptic sites. We have analyzed the role of Gopc by knockdown in primary cultured neurons and by generating a conditional Gopc knockout (KO) mouse line. In neurons, targeting of neuroligin 1 (Nlgn1) and metabotropic glutamate receptor 5 (mGlu5) to the plasma membrane was impaired upon depletion of Gopc, whereas NMDA receptors were not affected. In the hippocampus and cortex of Gopc KO animals, expression levels of Gopc-associated receptors were not altered, while their subcellular localization was disturbed. The targeting of mGlu5 to the postsynaptic density was reduced, coinciding with alterations in mGluR-dependent synaptic plasticity and deficiencies in a contextual fear conditioning paradigm. Our data imply Gopc in the correct subcellular sorting of its associated mGlu5 receptor in vivo.

**Supplementary Information:**

The online version contains supplementary material available at 10.1007/s12035-021-02504-9.

## Introduction

The targeting of membrane proteins to specialized locations on the cell surface is a complex and highly regulated process. This is especially true for membrane receptors of postsynaptic sites in neurons, which are mostly synthesized in the neuronal soma at ER-associated ribosomes and then need to be processed through Golgi compartments, before being transported along dendrites. There they are finally incorporated into postsynaptic membrane-associated protein complexes that, in the case of excitatory glutamatergic synapses, are localized on dendritic spines. In neurons, the Golgi apparatus is mostly found in the cell body in its usual, perinuclear position, but also may form extensions in some of the dendrites in the form of so-called Golgi outposts [1]. Besides, a dendritic Golgi satellite network has been observed which may assist in the processing of membrane proteins in dendrites [2]. Nevertheless, for most postsynaptic sites, the Golgi is relatively far away. Thus, it is unclear whether and how membrane receptors destined for dendritic spines and the postsynaptic density (PSD) are sorted in the Golgi.

One important determinant for the subcellular distribution and anchoring of postsynaptic membrane proteins is the presence of a so-called PSD-95/discs large/ZO-1 (PDZ) ligand motif at the C-terminus of many membrane receptors, which can bind to PDZ domains of various scaffold proteins at synapses [3]. This holds true, among others, for GluN2A/B subunits of NMDA receptors [4], the stargazin proteins which are involved in the anchoring of AMPA receptors [5], and for several subtypes of mGluRs. Also, cell adhesion molecules such as members of the Nlgn family may carry such a motif [6, 7]. Typical membrane-associated, PDZ domain containing scaffold proteins, which are involved in anchoring receptors, are members of the PSD-95 and Shank families of proteins. These are enriched in the postsynaptic density in a plasma membrane-proximal position to fulfill their functions.

An additional PDZ domain-containing protein, which binds to many postsynaptic membrane receptors, is the Golgi-associated PDZ and coiled-coil motif-containing protein (Gopc; also known as protein interacting specifically with Tc10, PIST; and as cystic fibrosis transmembrane conductance regulator (CFTR)-associated ligand, CAL). Gopc consists of two N-terminal coiled-coil regions, a linker region, and a C-terminal PDZ domain [8–11]. Gopc is widely expressed in many tissues; immunocytochemical analyses have consistently shown that the protein is localized at the trans-Golgi network (TGN) in different tissues and cell types, due to association of its coiled-coil regions with Rab6, syntaxin-6, and golgin-160 [10, 12–16]. Through the PDZ domain, Gopc interacts with a wide variety of transmembrane cell surface receptors, including CFTR, several G-protein coupled receptors including mGlu5, stargazin, neuroligins, and GluN2A/B subunits [6, 9, 10, 12, 17, 18]. As these receptors and ion channels fulfill their functions at the plasma membrane, whereas Gopc resides at the TGN, it is understood that Gopc interacts transiently with these membrane proteins during some sorting step either in the biosynthetic pathway or at a post-endocytic phase of receptor trafficking. However, which part of the sorting machinery specifically is affected by Gopc has been a subject of debate, as it may be receptor- and cell type-specific [18–20]. Thus, for the CFTR, it has been proposed that Gopc contributes to its degradation in lysosomes [8, 10, 21], whereas for mGlu5 it was reported that degradation of the receptor by the ubiquitin/proteasomal system is prevented by Gopc [22, 23]. A more common finding has been that overexpression of Gopc leads to retention of its associated membrane proteins at the TGN [12, 17, 24, 25]. However, as most of these results have been obtained in cellular systems where Gopc or its interaction partners have been overexpressed, we do not know yet whether Gopc regulates the abundance, stability, or subcellular targeting in an in vivo setting. As several of the membrane receptors which are believed to be regulated by Gopc contribute to the regulation of synaptic strength and to synaptic plasticity, this aspect is particularly interesting for our understanding of learning and memory processes.

We have asked here which synaptic membrane proteins are regulated by Gopc in the nervous system. For this, we have analyzed the effect of Gopc deficiency in cultured neurons upon *Gopc* knockdown, as well as in mouse brain after conditional knockout of the gene. We observe that the membrane receptor which is most strongly affected by the Gopc deficiency is the mGlu5, which is not properly targeted to the postsynaptic density in the hippocampus. This coincides with altered mGlu5-dependent long-term depression (LTD) and deficits in fear-related hippocampal memory in adult mice.

## Materials and Methods

### Antibodies

Primary and secondary antibodies used in this study are listed in Tables 1 and 2.

**Table 1:** Primary antibodies. *WB*, dilution for Western blotting; *IF*, dilution for immunofluorescence staining

Antibody	Host	Dilution	Reagent	Company
Akt	Rabbit	WB 1:1000	5% BSA/TBS-T	Cell Signaling (9272)
Phospho-Akt (pAKT) (Ser473)	Rabbit	WB 1:1000	5% BSA/TBS-T	Cell Signaling (9271)
Alpha-tubulin	Mouse	WB 1:1000	5% milk powder/TBS-T	Abcam (ab7291)
p44/42 (Erk 1/2)	Rabbit	WB 1:1000	5% milk powder/TBS-T	Cell Signaling (9102)
Phospho-p44/42 (pErk 1/2) (Thr202/Tyr204)	Rabbit	WB 1:1000	5% BSA/TBS-T	Cell Signaling (9101)
p38	Rabbit	WB 1:1000	5% BSA/TBS-T	Cell Signaling (8690)
Phospho-p38 (P-p38) (Thr180/Tyr182)	Rabbit	WB 1:1000	5% BSA/TBS-T	Cell Signaling (4511)
GluA1	Rabbit	WB 1:1000	5% milk powder/TBS-T	Alomone (AGC-004)
MAP2	Chicken	IF 1:1000	2% horse serum/PBS	Antibodiesonline (ABIN372661)
mGlu5	Rabbit	WB 1:1000	5% milk powder/TBS-T	Merck (ab5675)
Nlgn1	Mouse	WB 1:1000	5% milk powder/TBS-T	Synaptic System (129–111)
Nlgn2	Rabbit	WB 1:1000	5% milk powder/TBS-T	Synaptic System (129–203)
GluN1	Mouse	WB 1:1000	5% milk powder/TBS-T	Merck (MAB363)
GluN2A	Rabbit	WB 1:1000	5% milk powder/TBS-T	Novus Biologicals (NB300-105)
GluN2B	Rabbit	WB 1:1000	5% milk powder/TBS-T	Novus Biologicals (NB300-106)
Gopc	Rabbit	WB 1:1000	5% milk powder/TBS-T	Sigma-Aldrich (HPA024018)
Gopc	Guinea pig	WB 1:1000 IF 1:1000	5% milk powder/TBS-T 2% horse serum/PBS	Serum, Kreienkamp lab, UKE
Stargazin	Rabbit	WB 1:1000	5% milk powder/TBS-T	Merck (07–577)
Transferrin receptor (Tfrc)	Rabbit	WB 1:1000	5% milk powder/TBS-T	Abcam (ab84036)

**Table 2:** Secondary antibodies

Antibody	Host	Conjugate	Dilution	Company
α-Chicken	Goat	Alexa-Fluor 633	1:1000	Thermo Fisher Scientific Inc (A21103)
α-Guinea pig	Goat	Horse radish peroxidase	1:2500	ImmunoReagents, Inc (GtXGp-003-DHRPX)
α-Guinea pig	Goat	Alexa-Fluor 555	1:1000	Thermo Fisher Scientific Inc
α-Mouse	Goat	Horse radish peroxidase	1:2500	ImmunoReagents, Inc (GtXMu-003-E2HRPX)
α-Rabbit	Goat	Horse radish peroxidase	1:2500	ImmunoReagents, Inc (GtXRb-003-EHRPX)

### Tissue Culture

Rat hippocampi and cortices were prepared from embryonic rats (E18). After dissection and dissociation by treatment with trypsin, cells were plated in plating media (DMEM + 10% horse serum) on poly-l-lysine treated glass coverslips (hippocampus) or 6-well plates (cortex). After 1 day, media was removed and neurons were cultured with complete Neurobasal media containing 2% B27 supplement. Media and supplements were obtained from Thermo Fisher.

### Knockdown of Gopc Expression in Primary Cultured Rat Hippocampal and Cortical Neurons

Potential shRNA sequences against the mRNA coding for rat *Gopc* were tested initially in pSuper vector by cotransfection of pSuper plasmids with an expression vector for Gopc in HEK-293 cells. The most efficient sequence (5’-GGCGGACATCACTTATGAGTTCAAGAGACTCATAAGTGATGTCCGCC-3’; reverse complementary sequences are underlined) was then subcloned into the lentiviral shRNA vector pLVTHM. This vector allows for coexpression of EGFP to identify transfected or infected cells. Vectors containing scrambled sequences, as well as an unrelated sequence were used as negative controls with similar results. Lentiviral particles were produced by cotransfection of pLVTHM-based plasmids with pSPAX2 (virus packaging) and pMDg2 (coding for VSV-G protein) into HEK-293 T cells. Viral supernatants were enriched using Lenti-X Concentrator (Clontech) and used for infection of cortical neurons.

### Immunocytochemistry on Hippocampal Neurons

Hippocampal neurons cultured on glass cover slips were fixed with 4% PFA in PBS and permeabilized with 0.1% TX-100. After blocking non-specific binding sites with 2% horse serum, the samples were immunostained. The coverslips were mounted on glass slides with ProLong Diamond Antifade Mountant with DAPI (Thermo Fischer) and analyzed by laser scanning confocal microscopy (Leica SP5).

### Biotinylation Experiments on Cortical Neurons

Cortical neurons cultured on 6-well plates were washed three times in ice-cold PBS supplemented with 0.1 mM CaCl_2_ and 1 mM MgCl_2_ (PBS-Ca-Mg) and biotinylated using EZ-Link Sulfo-NHS-SS-Biotin (Thermo Scientific; 1 mg/ml) in the same buffer (30 min; 4 °C). After quenching excess reagent with 100 mM glycine in PBS-Ca-Mg, cells were lysed in RIPA buffer (150 mM NaCl; 50 mM Tris–HCl, pH 8.0; 5 mM EDTA; 1% NP-40; 0.5% sodium deoxycholate; 0.1% SDS), followed by centrifugation at 20,000 × g (20 min; 4 °C). Biotinylated proteins from two wells per sample were purified from the supernatant with NeutrAvidin Agarose beads (Thermo Fisher; 4 °C, overnight, under rotation), followed by extensive washing with RIPA buffer. Samples of lysate and precipitate were incubated in Laemmli buffer (4 °C, overnight) and analyzed by Western blotting.

### DHPG Stimulation

Infected cortical neurons cultured on 6-well plates were treated with an inhibitor cocktail (1 µM tetrodotoxin (TTX, Bio Trend), 40 µM 6-cyano-7-nitroquinoxaline-2,3-dione (CNQX, Sigma-Aldrich), 100 µM 2-amino-5-phosphonopentanoic acid (AP-5, Tocris), and 5 µM nimodipine (Sigma-Aldrich)) for 4 h to inhibit/reduce synaptic signaling. This was followed by treatment with 100 µM (RS)-3,5-dihydroxyphenylglycine (DHPG, Tocris) for 5 min under inhibitory conditions. Cells were lysed in RIPA buffer, and lysates were mixed with Laemmli buffer and analyzed by Western blotting.

### Generation of Conditional Gopc KO Mice

A mouse embryonic stem cell clone (EPD0822_2_B04) carrying an exon trap cassette in the mouse *Gopc* gene was obtained from the European Mouse Mutant Cell Repository (EuMMCR) at Helmholtz-Zentrum in Munich, Germany. Generation of heterozygous mice (C57BL/6-Gopc^Tm1a^ line) has been described previously [19]. The modified *Gopc* allele in these mice is not functional, due to an artificial splice acceptor (exon trap; so-called KO first configuration). In this line, we could generate only very few homozygous, Gopc-deficient animals, probably due to a high proportion of embryonic lethality in homozygous KO animals [19]. For generation of conditional KO mice, we crossed with a Flp-deleter line [26], leading to the *Gopc*^Tm1c^ allele, where the critical exon 3 is flanked by LoxP sites. Forebrain specific deletion of this exon (in the *Gopc*^Tm1d^ allele) was then achieved by crossing with a strain of mice expressing Cre under control of the αCaMKII promoter [27]. Mouse genotyping and the occurrence of the desired correct recombination events were in each case verified on the genomic level by PCR.

All mice were cared for and treated strictly following the ethical animal research standards defined by the Directive of the European Communities Parliament and Council on the protection of animals used for scientific purposes (2010/63/EU). Experiments with mice described in this manuscript were approved by local ethics committees. In particular, all experimental procedures in Hamburg were approved by Behörde für Gesundheitsschutz und Verbraucherschutz of the Freie und Hansestadt Hamburg, Germany under applications 90/14 and 81/12; experiments in Magdeburg were approved by the Ethical Committee on Animal Health and Care of Saxony-Anhalt state, Germany (license number: 42502–2-1346).

### Tissue Lysates

Tissues were prepared and immediately frozen in liquid N_2_. For preparation of lysates, tissues were homogenized in RIPA buffer using a potter, followed by incubation for 30 min on ice. Insoluble material was removed by centrifugation (20,000 × g; 4 °C). Supernatants were processed for Western blotting by adding Laemmli buffer.

### Enrichment of the Hippocampal PSD Fraction

Enrichment of the hippocampal PSD fraction was performed based on a procedure described by Coba et al. [28], which uses differential extraction with detergents. Hippocampi were homogenized in 10 mM HEPES buffer pH 7.4, containing 2 mM EDTA, 5 mM sodium orthovanadate, 30 mM NaF, 20 mM β-glycerol phosphate, and Roche Complete protease inhibitor cocktail. After centrifugation for 5 min at 500 × g, the supernatant was collected. Pellets were extracted again, followed by centrifugation at 500 × g. Combined supernatants were centrifuged at 10,000 × g, and the membrane pellet was solubilized in homogenization buffer supplemented with 1% Triton X-100. Solubilized membranes were centrifuged at 30,000 × g for 40 min. Both the pellet and supernatant (soluble) fractions of this step were solubilized in Laemmli sample buffer at 4 °C overnight, without boiling. PSD enrichment was verified by Western blotting, showing a strong enrichment of the postsynaptic scaffold proteins of the Shank family in the Triton-insoluble pellet.

### Preparation of P2 Membrane Fraction

Forebrains isolated from adult mice were mechanically homogenized in 4 mM Hepes, 0.32 M saccharose, 1 mM MgCl_2_, 0.5 mM CaCl_2_, pH 7.4, and protease and phosphatase inhibitors cocktails. After several low speed centrifugation steps (1500 × g) to pellet the nuclei and cell debris, a postnuclear supernatant was centrifuged at 13,800 g (4 °C, 15 min) to obtain the P2 membrane fraction.

### Electrophysiological Analysis

Hippocampal slices were acutely prepared from 4- to 6-month-old conditional Gopc mice as previously described [29]. Mice were killed by decapitation and the removed brain was placed into ice-cold solution containing (in mM) 240 sucrose, 2 KCl, 1 MgCl_2_, 2 MgSO_4_, 1 CaCl_2_, 1.25 NaH_2_PO_4_, 26 NaHCO_3_, and 10 glucose (osmolarity of 300 ± 5 mOsm). Transverse hippocampal slices (350 μm) were obtained from the left hippocampus [30] and kept in a submerged chamber until transfer to a submerged recording chamber, supplied continuously with artificial cerebrospinal fluid (ACSF) solution containing (in mM) 124 NaCl, 5 KCl, 2 CaCl_2_, 1 MgSO_4_, 26 NaHCO_3_, 1.25 NaH_2_PO_4_, and 10 glucose (osmolarity of 290 ± 5 mOsm). All solutions were saturated with 95% O_2_/5% CO_2_.

Field excitatory postsynaptic potentials (fEPSPs) were recorded with low-resistance glass electrodes (Hilgenberg, Germany) filled with ACSF. A stimulating electrode was placed in the stratum radiatum of CA1 to stimulate the Schaffer collateral pathway. A recording electrode (~ 2 MOhm) was about ~ 300 μm apart from the stimulating one. Using stimulus isolator (A385, WPI, USA), the input–output curve was determined for the analysis of basal synaptic transmission. After a stable baseline recording of fEPSPs for 10 min, DHPG (100 μM, Tocris) was applied for 10 min and then washout with drug-free ACSF solution. Electrophysiological data were recorded with Patchmaster (Heka Elektronik, Germany) and then off-line analyzed with Sigmaplot 12.3 (Systat software, USA), Prism7 (GraphPad, USA), and pClamp10 (Molecular Devices, USA). Paired-pulse facilitation was expressed in % as the ratio between the slopes of the second and first responses evoked by paired stimulation. LTD was expressed as the average change in % from baseline ± SEM. The data were presented as mean ± SEM.

### Behavioral Analysis

Adult mice (14 females and 15 males for each genotype) were subjected to a battery of standard behavioral tests, namely, the open field and elevated plus maze test for novelty-seeking and anxiety and the spontaneous alternation for working memory, the water maze for spatial learning and memory, and the contextual fear conditioning for emotional memory. The open field test was performed in a box (50 × 50 cm and 40-cm high) illuminated with white light (100 lx). Mice were started from one corner of the box, and their behavior was analyzed for 20 min. Distance moved and mean minimal distance to the wall were analyzed with the software EthoVision (Noldus, Wageningen, The Netherlands). The elevated plus maze had the shape of a plus with four 30-cm long and 5-cm wide arms, connected by a squared center (5 cm × 5 cm). Two opposing arms were bordered by 15-cm high walls (closed arms), whereas the other two arms (open arms) were bordered by a 2-mm rim. The maze was elevated 75 cm from the floor and an infrared camera allowed video-recording under total darkness. The mouse was placed into the center facing one open arm and left on the maze for 5 min. Time spent in the different arms was analyzed with the software The Observer (Noldus). The spontaneous alternation test was done to test for working memory performance [31]. The maze consisted of three equally sized arms (34 cm × 5 cm × 30 cm) made of transparent Plexiglas connected such as to make a Y and illuminated with 5 lx. Mice were placed in the center of the maze and allowed to freely explore the maze until they performed 27 transitions or after a maximal given time of 20 min. An entry into any arm with the four paws was considered a transition. An entry into a new arm after having visited the two other arms was considered as alternation. Data were analyzed as a percentage of alternations over all transitions.

The water maze consisted of a circular tank (145 cm in diameter) circled by dark curtains. The water was made opaque by the addition of non-toxic white paint such that the white platform (14-cm diameter, 9-cm high, 1-cm below water surface) was not visible. Four landmarks (35 × 35 cm) differing in shape and gray gradient were hung on the wall of the maze. The light was provided by four white spotlights placed on the floor around the swimming pool that provided homogeneous illumination of 60 lx on the water surface. Before the experiment started, mice were familiarized for 1 day to swim and climb onto a platform (diameter of 10 cm) placed in a small rectangular maze (42.5 × 26.5 cm and 15.5-cm high). During familiarization, the position of the platform was unpredictable since its location was randomized and training was performed under darkness. After familiarization, mice underwent 3 learning days during which they had to learn the location of a hidden platform. The starting position and position from which mice were taken out of the maze were randomized. At days 1 and 2, mice underwent four learning trials (maximum duration 90 s, inter-trial interval of 10 min). On day 3, mice underwent 2 learning trials. After staying on the platform for 15 s, mice were returned to their home cage and warmed up under red light. Mice underwent three 60-s long transfer trials during which the platform was removed and time spent in four imaginary quadrants was measured with EthoVision. The first transfer trial was done on day 2, 20 min after the second learning trials (for short-term memory); the second transfer trial was done 24 h after the last learning trial of day 2 and before the first learning trial on day 3 (for long-term memory); and the third transfer trial was done 7 days after the last learning trial on day 3 (for remote memory).

In the contextual fear conditioning test, mice had to learn the association between the unconditioned (electric footshock) and conditioned stimuli (context). Mice were conditioned in the context, a chamber (23.5 × 23.5 cm and 19.5-cm high) with Plexiglas walls and ceiling and a stainless grid floor from which an electric shock could be elicited. The chamber was illuminated by white light (45 lx). Mice were placed in the center of the cage and received three electric footshocks (0.35 mA, 1 s) at 120, 160, and 200 s. At 240 s, the recording ended, and the mouse was immediately returned into its home cage. Twenty-four hours after conditioning, mice underwent a recall trial during which they were placed again in the context for 3 min. Twenty-four hours after the recall trial, we tested generalization of the fear response by placing the mouse in a new box (30 × 15 cm and 25-cm high) made of white PVC for 3 min: it is expected that mice discriminate between the different environments and thereby spend more time freezing in the conditioned context than in the new box. We thus calculated the following discrimination index: % of time freezing in the conditioned context − % of time freezing in the new box. The conditioned response was analyzed by quantifying the percentage of time spent freezing (defined as the absence of body movements for at least 1 s). Freezing behavior was automatically analyzed using a modified version of the system Mouse-E-Motion (Infra-e-motion, Hamburg, Germany).

For all tests, no mice/no data point were excluded from the analysis. All mice underwent all behavioral tests with an interval of at least 2 days between tests. Experiments and analyses were done by an experimenter blind to the genotype, in agreement with ARRIVE guidelines.

### Statistics

Basal synaptic transmission (stimulus–response curves) was analyzed using repeated two-way ANOVA. Blot data, as well as electrophysiological data (paired-pulse facilitation and long-term depression) were compared between genotypes using the unpaired *t*-test, or one-way ANOVA, as indicated. All behavioral data were analyzed by two-way analysis of variance with genotype and sex as between-group factors. All tests were two-tailed and the level of significance was set at *p* < 0.05.

## Results

**Fig. 1:**
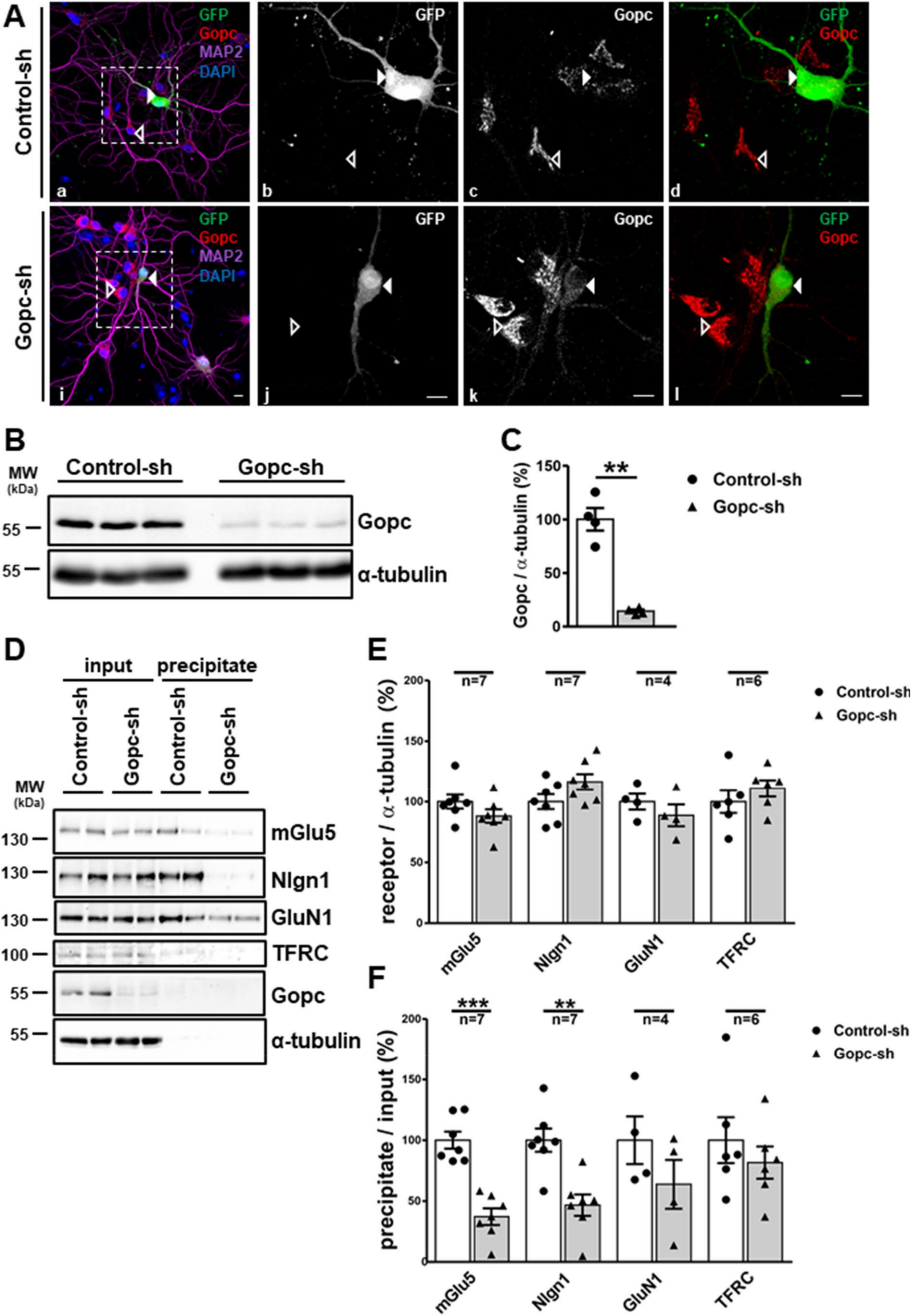
**A** Hippocampal neurons were transfected with an shRNA control vector, or shRNA vector directed against *Gopc*. Cells were stained using GFP (expressed from the pLVTHM shRNA vector), to identify transfected cells, the dendritic marker MAP2, and Gopc. Nuclei were detected with DAPI. Transfected cells are labeled with an arrowhead. **B** Cultured cortical neurons were infected with lentiviral particles coding for control or *Gopc*-specific shRNA; cells were lysed and analyzed by Western blotting using the antibodies indicated. **C** Quantification of the data shown in **B**; Gopc levels were normalized to α-tubulin levels. **D**–**F** Infected neurons as prepared in **B** were subjected to surface biotinylation. Biotinylated proteins were precipitated from cell lysates, and input and precipitate samples were analyzed by Western blotting. Quantification in **E** was performed by normalization to α-tubulin levels; in **F**, surface receptors were normalized to the total amount of receptors present in cellular lysates. **, significantly different from control, *p* < 0.01; *t*-test; *n* = 4–7. Mean + SEM values are shown

For the analysis of Gopc function in neurons, we developed an shRNA knockdown of *Gopc* in primary cultured rat neurons. Various shRNA sequences were initially tested against a rat *Gopc* expression construct upon cotransfection in HEK-293 T cells. The sequence providing the most efficient knockdown at the Western blot level was then subcloned into the pLVTHM vector that allows for production of lentiviral particles, and which also carries a GFP expression cassette for the identification of transfected or infected cells. Transfection of this construct into primary cultured hippocampal neurons, followed by immunostaining using anti-Gopc antisera, showed the typical Golgi-like appearance of Gopc in non-infected neurons and a complete loss of staining in GFP-positive transfected cells (Fig. 1A). Viral particles were then produced from this vector and used for infection of primary cortical neurons. Cultures that showed a high percentage of infected (GFP-positive) cells were analyzed by Western blotting, using anti-Gopc and α-tubulin antibodies. Here, we observed a residual amount of Gopc protein of 14% compared to neurons infected with a control virus derived from pLVTHM empty vector (Fig. 1B,C).

We then asked how the deficiency in Gopc affected the abundance and the subcellular sorting of its interaction partners. For this, the infected cortical neurons were treated with a non-membrane permeable biotinylating reagent. Cells were then lysed and biotinylated proteins were isolated using streptavidin beads. Samples from cell lysates as well as from precipitates were analyzed by Western blotting using antibodies against various Gopc-interacting membrane proteins; we focused mostly on abundant postsynaptic proteins for which a known interaction of a C-terminal PDZ domain ligand with the PDZ domain of Gopc had been published. In addition, we included stargazin, which binds through an upstream sequence to the linker region of Gopc [18]. Furthermore, we included the transferrin receptor (Tfrc), which does not carry a C-terminal PDZ ligand motif and is not known to interact with Gopc, as a negative control. We observed that the total cellular content of all proteins tested remained unchanged after *Gopc* knockdown, indicating that Gopc is not involved in either degradation or stabilization of its interaction partners (Fig. 1C). However, we observed a significant reduction of mGlu5 and neuroligin-1 in the biotinylated (i.e., cell surface-associated) fraction, indicating that for these two proteins Gopc is necessary for efficient targeting or anchoring at the cell surface. NMDA receptors have been suggested to interact with Gopc through their GluN2A or GluN2B subunits [18]; however, the amount of NMDA receptors (measured via the abundance of the GluN1 subunit) was not changed in either total lysate or in the biotinylated fraction. Furthermore, the transferrin receptor was also not affected by *Gopc* knockdown (Fig. 1D,F).

**Fig. 2:**
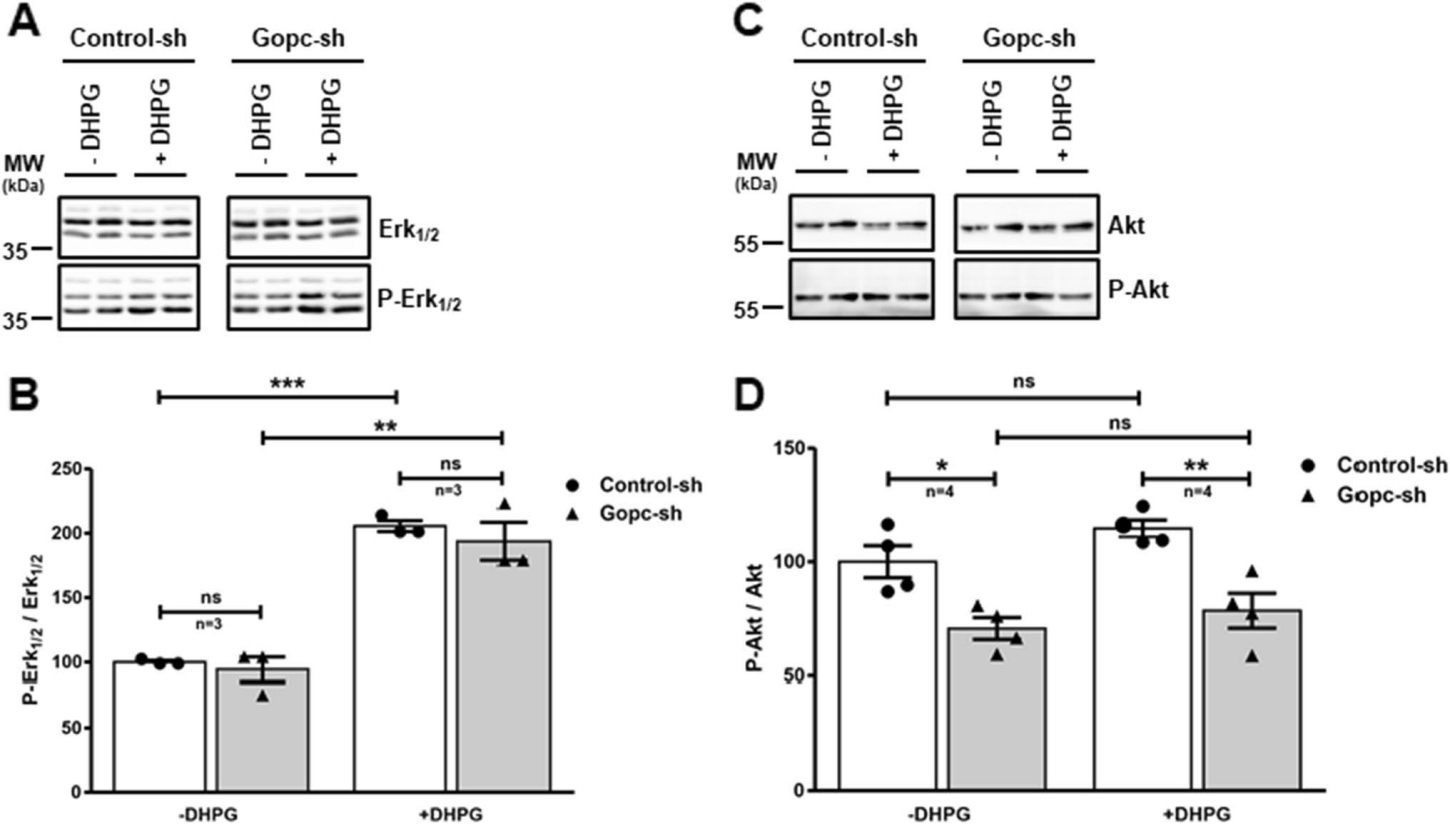
Reduced Akt signaling after knockdown of *Gopc* in cortical neurons. Primary cortical neurons were infected with lentiviral particles carrying *Gopc* shRNA, or control shRNA. Neurons were treated with an inhibitor cocktail for 4 h to inhibit/reduce synaptic signaling followed by treatment with 100 µM DHPG for 5 min under inhibitory conditions. Cell lysates were mixed with Laemmli buffer and analyzed by Western blotting. Data are presented as the ratio of phospho-kinase to total kinase signal, normalized to unstimulated control conditions. *, **, *** significantly different from control, *p* < 0.05, 0.01, 0.001, respectively. One-way ANOVA, followed by Tukey’s post hoc comparison; *n* = 3. Mean + SEM values are shown

As mGlu5 leads to activation of signaling pathways involving Erk_1/2_ and Akt kinases, we treated cultures with the mGluR1/5 specific agonist DHPG. Here, we observed that Erk_1/2_ signaling, measured as the ratio of pErk_1/2_ to total Erk_1/2_, was not altered upon loss of Gopc expression (Fig. 2A,B). In these experiments, the Akt pathway was not noticeably activated upon mGluR1/5 activation. However, basal levels of Akt phosphorylation were significantly decreased in *Gopc* knockdown neurons (Fig. 2C,D).

**Fig. 3:**
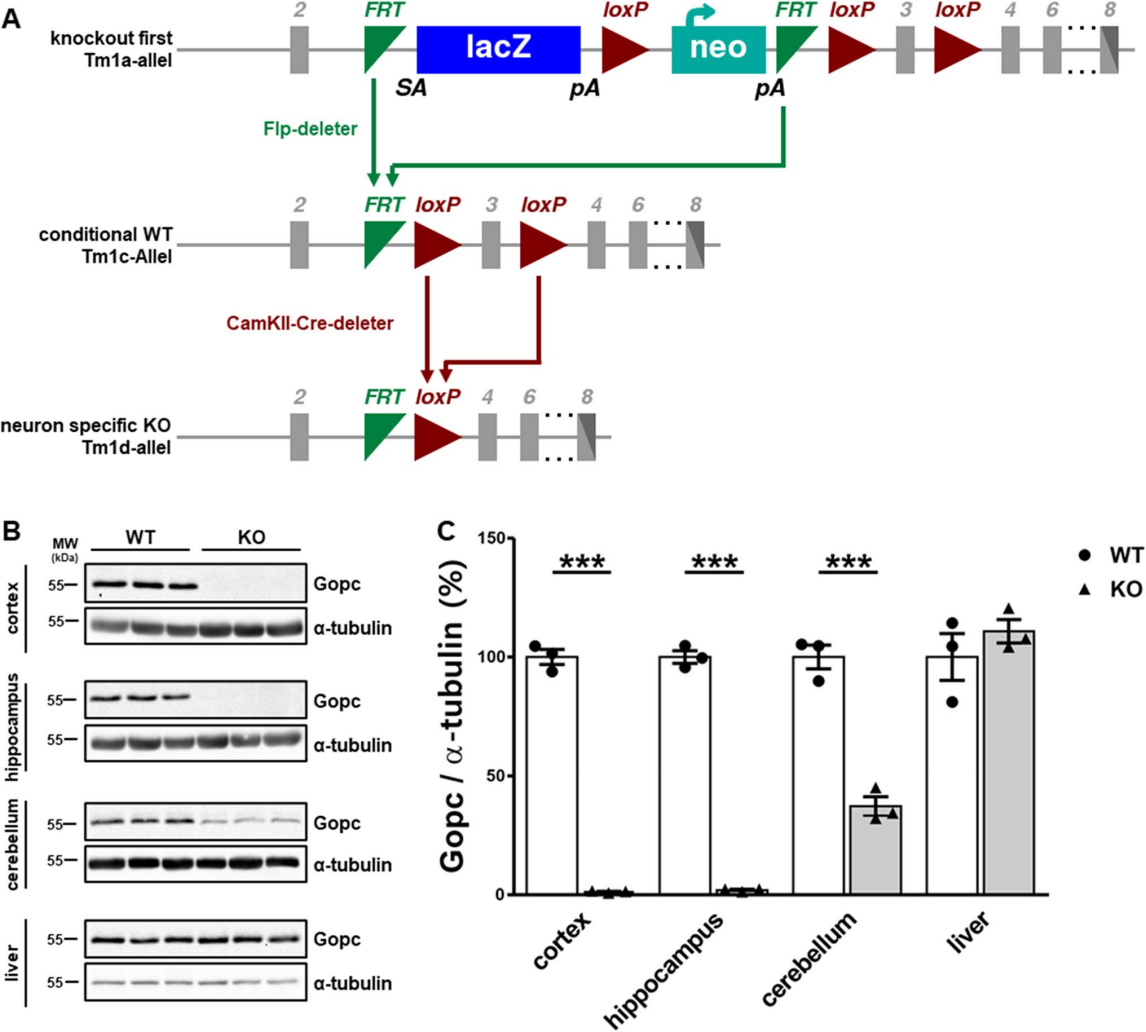
Conditional knockout of *Gopc* in mouse forebrain neurons. **A** Strategy for conditional inactivation of the mouse *Gopc* gene. Mice carrying the non-conditional “knockout first” allele were first crossed with a Flp deleter mouse, to remove the LacZ/neomycin cassette, which was used for targeting the *Gopc* gene. In a second step, mice homozygous for this conditional allele were mated with mice expressing Cre recombinase under the control of the αCaMKII gene promoter, leading to recombination via LoxP sites as indicated. Exon 3 of the *Gopc* gene is critical, and functional protein cannot be expressed in its absence. **B + C** Verification of loss of Gopc expression in various brain regions, as analyzed by Western blot of tissue lysates. In agreement with the known expression pattern of the αCaMKII, we observed complete loss of Gopc in the hippocampus and cortex, moderate loss in the cerebellum, and no change in the liver. ***, significantly different from control, *p* < 0.0001; *t*-test; *n* = 3. Mean + SEM values are shown

**Fig. 4:**
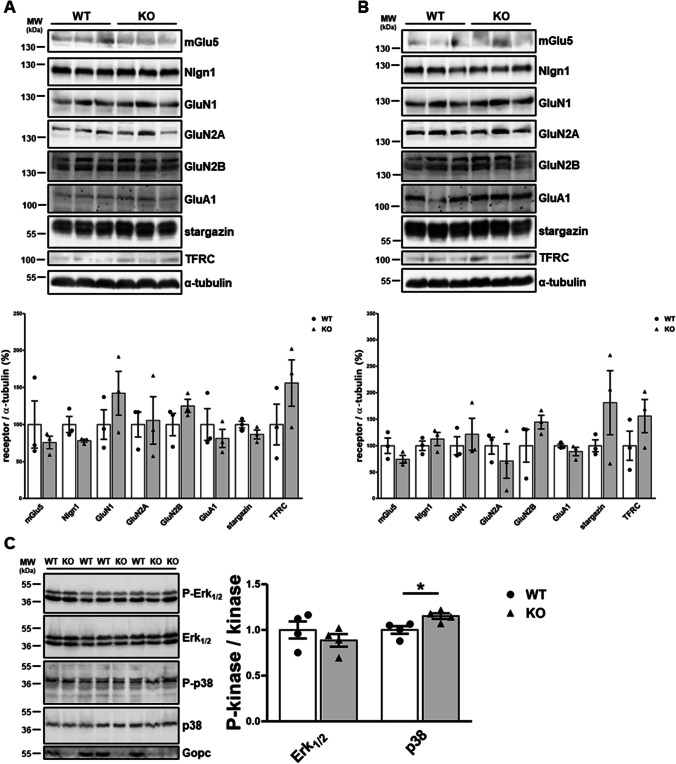
Loss of Gopc does not affect the abundance of its associated receptors. **A**–**B** Lysates from the hippocampus (**A**) and cortex (**B**) isolated from wt and conditional Gopc-deficient (KO) mice were analyzed by Western blotting using antibodies against a number of membrane proteins, as indicated. For quantification, signal strength was in each case normalized against α-tubulin levels. In both tissue types, no significant changes between WT and KO were detected; *n* = 3. **C** Hippocampal lysates from both genotypes were analyzed by Western blotting using antibodies against phosphorylated (active) and total forms of signaling kinases. The kinase activity was quantified as the ratio of phosphorylated to the total kinase levels. *, statistically significant difference, *p* < 0.05; *t*-test; *n* = 4. Mean + SEM values are shown

As a second experimental system, we established a Gopc-deficient mouse line. We had previously described *Gopc* KO mice which had been generated by homologous recombination [19]. These carry a selection cassette which leads to the inactivation of the mouse *Gopc* gene in all tissues (in the so-called KO first approach). However, from this line of mice, we could only generate a very limited number of homozygous Gopc-deficient animals, as we obtained less than 2% of KO animals from heterozygous breedings [19]. Therefore, we made use of FRT and LoxP recombination sites present in the targeting cassette which was used for the generation of these mice. By first crossing with mice expressing Flp recombinase, we created a line with a *Gopc* allele which is functional but which can be deleted by expression of Cre recombinase (Fig. 3A). By crossing with a mouse line that expresses Cre under the control of the promoter for the α-subunit of Ca^2+^/calmodulin-dependent kinase II (αCaMKII) [27], we obtained mice with postnatal loss of *Gopc* expression in specific brain areas, including cortex, hippocampus, and amygdala. Expression of Cre recombinase has also been reported in thalamus, hypothalamus, and striatum, but neither in cerebellum nor any peripheral tissues. Successful knockout was verified by Western blotting of various tissues from these mice, where we observed almost complete loss of Gopc immunoreactivity in the hippocampus and cortex, a partial loss in the cerebellum, and no change in the liver (Fig. 3B). We isolated cortical and hippocampal tissue from Cre-expressing (KO) and non-Cre-expressing (WT) animals and analyzed protein extracts of these tissues again by Western blotting using antibodies against Gopc-interacting proteins (Fig. 4A,B). Here, we observed again no quantitative changes in the levels of all proteins analyzed, confirming our previous observation that the deficiency in Gopc does not affect steady-state levels of its associated membrane receptors. Part of this analysis was repeated with a membrane fraction isolated from forebrains of mice; again no differences were observed (Supplemental Figure S1). Furthermore, we analyzed signaling proteins of the different MAP kinase cascades, as it is well known that Gopc-associated proteins like mGlu5 or NMDA receptors are involved in the activation of these cascades. The activity was in each case measured as the ratio between the amount phosphorylated/active form of the kinase and the total amount of the kinase. Here, we saw no change in the activity of Erk_1/2_ kinases, but a rather moderate but significant increase in p38 MAP kinase activity in the hippocampus of *Gopc* KO mice (Fig. 4C). No change was observed for Akt activity in total forebrain lysates (Supplemental Figure S2).

**Fig. 5:**
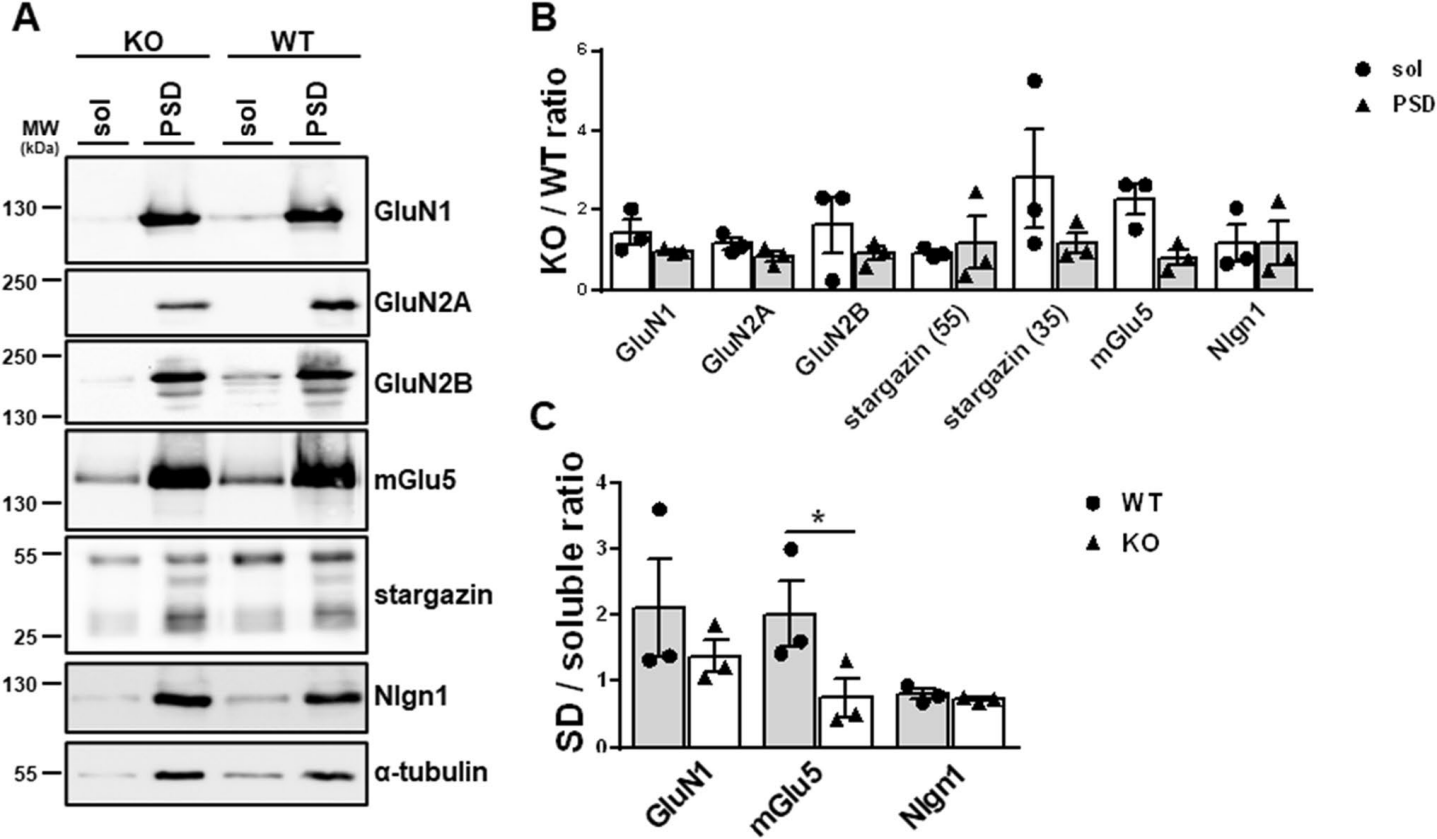
Loss of mGlu5 in the postsynaptic fraction of *Gopc* KO mice. Hippocampi from KO and WT mice were subjected to a differential detergent extraction protocol which leads to a soluble (sol) fraction and a postsynaptic density (PSD) fraction (see “Materials and Methods” section for details). **A** These fractions were analyzed by Western blotting using the antibodies indicated; signal strength was in each fraction normalized to the α-tubulin signal. **B**–**C** Quantifications show the ratio of receptor amounts from KO to WT animals (**B**) and the ratio between PSD-associated vs soluble protein for a number of selected receptors (**C**). For stargazin, 55 kDa and 35 kDa bands were observed in **A** and separately evaluated in **B**. Note that the relative proportion of mGlu5 in the PSD fraction is significantly reduced in KO animals. *, statistically significant difference, *p* < 0.05; *t*-test; *n* = 3. Mean + SEM values are shown

Several of the Gopc-interacting membrane proteins are components of the postsynaptic density (PSD) of glutamatergic synapses. To address whether targeting of these proteins to the PSD is altered in the absence of Gopc, we isolated a PSD-enriched fraction from the hippocampi of WT and Gopc-deficient animals. By comparing the amount of PSD-associated interaction partners of Gopc to the total amount present in the hippocampal lysates, we determined that the expression of most receptors was not altered. However, we saw a clear and significant decrease in the amount of mGlu5 targeted to the PSD (Fig. 5). Taken together with the data from primary cultured neurons (Fig. 1), we, therefore, identify mGlu5 as the receptor that is most strongly affected by a loss of *Gopc* expression.

**Fig. 6:**
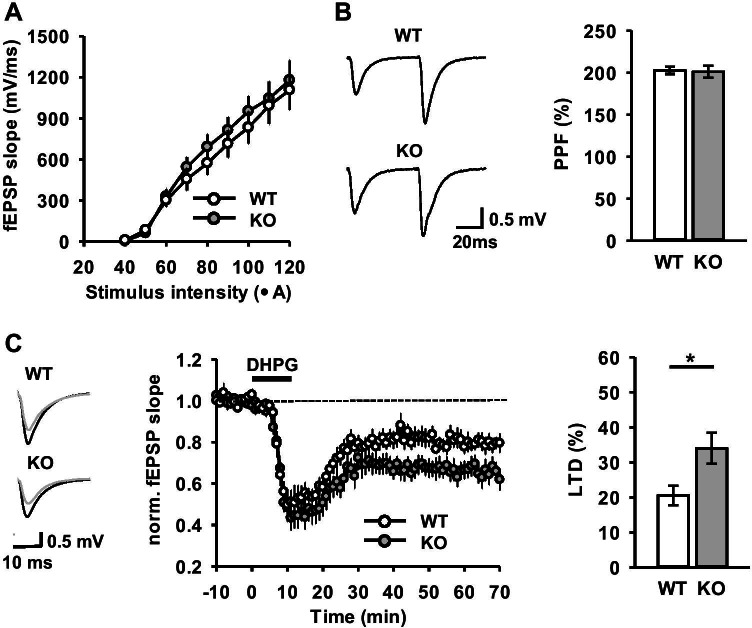
Enhancement of DHPG-induced hippocampal LTD in *Gopc* KO mice. **A** Input–output relationship for CA3-CA1 synapses in WT and KO mice. **B** (left) Representative traces of fEPSPs evoked by paired-pulse stimulation with 50-ms inter-stimulus interval. (right) Summary data of paired-pulse facilitation (PPF) recorded with 50-ms interval show no differences between WT and KO mice. **C** (middle) Application of DHPG (100 μM for 10 min, shown by a bold horizontal bar) leads to LTD in the hippocampal CA1 area. (left) Insets show representative traces of fEPSPs before and 60 min after DHPG application. The dashed line indicates a baseline level. (right) Summary diagram of DHPG-induced LTD (last 10 min of recordings) shows statistically significant difference between genotypes (*p < 0.05; *t*-test). WT, *n* = 6–8 slices; KO, *n* = 8–10 slices. Mean ± SEM values are shown

To characterize the effects of Gopc deficiency on synaptic function, we performed extracellular fEPSP recordings in acutely prepared hippocampal slices from WT and *Gopc* KO mice. Analysis of the input–output relationship between the fEPSP slope and stimulus intensity suggests that basal synaptic transmission was preserved intact in the Gopc-deficient hippocampal network (effect of genotype: *F*_1,12_ = 0.280, *p* = 0.6; Fig. 6A). Presynaptic function determined by recordings of paired-pulse facilitation (PPF) was also not different between the two groups (*t*_15_ = 0.176, *p* = 0.863; Fig. 6B). On a functional level, mGlu5 is well-known for its contribution to long-term depression (LTD) of glutamatergic transmission in the hippocampus. This form of hippocampal plasticity can be induced by activation of mGlu5 with its agonist, DHPG. Ten minutes of DHPG (100 μM) application resulted in ~ 20% LTD in WT slices 1 h after induction. Interestingly, LTD in KO slices was significantly augmented to ~ 35%, as compared with WT (*t*_16_ = 2.425, *p* = 0.027; Fig. 6C).

**Fig. 7:**
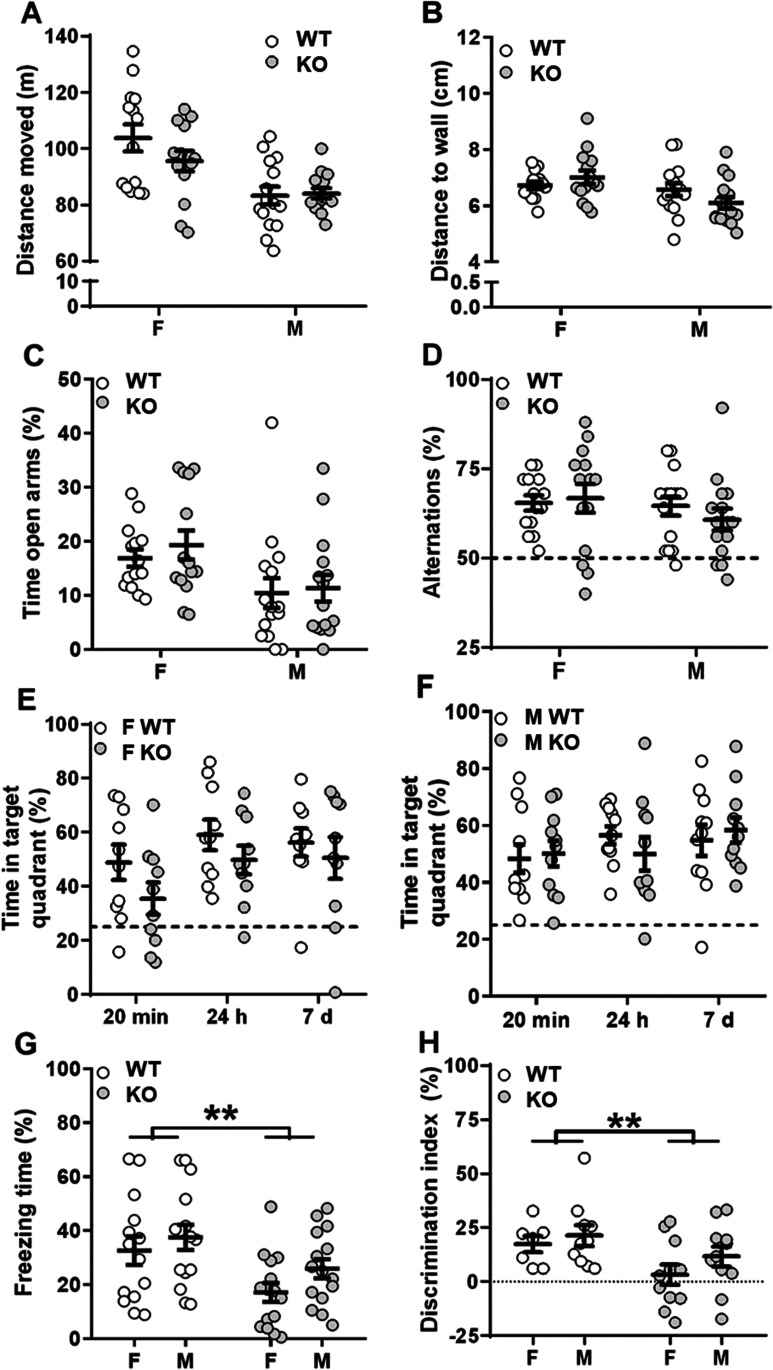
Impaired contextual fear conditioning in Gopc-deficient mice. **A**–**B** No differences were detected between genotypes in distance moved (**A**) and mean minimal distance to the wall (**B**) during the 20-min open field test. **C** WT and KO littermates spent equal amount of time on the open arms of the elevated plus maze. **D** Both genotypes had alternation rates above the chance level of 50% (indicated with a dotted line) in the spontaneous alternation test indicating that working memory was not affected by the genotype. **E**–**F** No differences between genotypes were detected in the water maze test. Female (**E**) and male (**F**) mice of both genotypes spent more time at the target quadrant than expected by chance (25%, indicated with a dotted line) during the transfer trials for short-term (24 min), long-term (24 h), and remote (7 days) memory. **G** Both female and male KO mice spent less time freezing during the recall trial of the contextual fear conditioning test as compared to WT littermates. **H** Both female and male KO mice had lower discrimination indexes than WT littermates indicating. F, female; M, male. **p < 0.01, the effect of genotype by 2-way ANOVA with genotype and sex as between groups factors; 14 females and 15 males per genotype. Mean + SEM values are shown

Given these changes in synaptic plasticity, we expected behavioral deficits in KO mice and performed a series of behavioral assays. Novelty-induced behavior and anxiety did not differ between genotypes as assessed in the open field (Fig. 7A,B) and elevated plus maze (Fig. 7C) tests. In the spontaneous alternation test, *Gopc* KO mice had a preference for the less familiar arm and performed as well as control littermates (Fig. 7D), suggesting that working memory is intact under Gopc deficiency. No differences between genotypes were detected in the water maze test: both genotypes learned to quickly find the hidden platform (data not shown) and showed similar performances in the transfer trials for short-term, long-term, and remote memory (Fig. 7E,F). However, *Gopc* KO mice showed significant deficits in the contextual fear conditioning paradigm. In this test, mice are introduced into a novel environment where they receive mild electrical footshocks. When reintroduced into the same environment on the next day, WT mice typically respond by freezing for prolonged times, as was also observed here. In contrast, male and female *Gopc* KO mice spent less time freezing as compared to WT mice during this memory retrieval period (effect of genotype: *F*_1,55_ = 10.1, *p* = 0.002; Fig. 7G). Moreover, contrary to control littermates, *Gopc* KO mice did not discriminate between the conditioned context and a novel context (effect of genotype: *F*_1,35_ = 6.1, *p* = 0.012; Fig. 7H). This indicates that *Gopc* KO animals did not learn or memorize the association between the electrical shocks and the context (i.e., the conditioning chamber). We detected no difference between genotypes during the 2 min after mice received the first footshock (FEM WT = 4.9 ± 1.6; FEM KO = 6.6 ± 3.2; MALE WT = 20.1 ± 5.6; MALE KO = 19.8 ± 5.7), indicating that anxiety toward the context and pain sensitivity, respectively, are unchanged in *Gopc* KO mice.

## Discussion

Gopc has been suggested to play diverse roles with respect to the membrane receptors that may associate with its single PDZ domain, ranging from acceleration of degradation to protection from degradation, to retention at the trans-Golgi network. Here, we used two experimental systems to assess the effects of Gopc deficiency on its associated membrane receptors. In primary cultured neurons, knockdown of Gopc expression by shRNA does not affect the abundance of its associated membrane proteins (Nlgn1, NMDA receptors, and mGlu5 were tested here). Instead, for two verified interaction partners of Gopc, namely, mGlu5 [22, 23] and Nlgn1 [6, 32], we observed that knockdown of Gopc reduces the amount of protein that is present at the cell surface. In the second set of experiments, we used a mouse line with a conditional deletion of the *Gopc* gene coding for Gopc in mouse forebrain neurons. Here again, we observed that in hippocampal and cortical tissues, levels of Gopc-associated membrane proteins were unchanged. Taken together, these data do not indicate that Gopc accelerates the degradation of interaction partners of its PDZ domain, as has been suggested for instance for the CFTR [21, 33]. It is likely that additional interaction partners, e.g., for the CFTR are required to cooperate with Gopc to target the CFTR to lysosomes. For mGlu5, it was observed that Gopc protects this receptor against proteasomal degradation as it interferes with its ubiquitination. mGlu5 is ubiquitinated by the E3 ligase Siah1 upon binding to the C-terminal tail of the receptor, and it was argued that Gopc might block access of the E3 ligase, thereby preventing receptor degradation [23, 34]. However, as we did not observe any reduction in the total levels of mGlu5 in *Gopc* KO mouse tissue, this might not be the major function of Gopc. Instead, in both our experimental systems, we observed that the mGlu5 was not targeted to its proper destination (towards the cell surface in cortical neurons and the postsynaptic density in the hippocampus) upon loss of Gopc. This is initially counterintuitive, as Gopc is not associated with the plasma membrane and is not a component of the PSD. Thus, it is not likely that Gopc acts as a scaffold or anchoring platform for mGlu5 at postsynaptic sites. We rather assume that Gopc maintains a role at the TGN (where it is localized) in sorting receptors to its final destination. For all interaction partners of the Gopc PDZ domain, there is at least one more PDZ domain protein which binds to the respective receptor at the plasma membrane (e.g., PSD-95 in case of Nlgn1 and stargazin; NHERF2 in the case of the mGlu5) [5, 7, 35]. Gopc may cooperate with these other PDZ domain proteins in some sort of network activity, as was described already in the case of the G-protein coupled receptor SSTR5 [20]. Thus, while Gopc may interact transiently with the receptors during the biosynthetic pathway, it may ensure that it is targeted to its plasma-membrane-associated PDZ partner.

In neurons, the decrease in membrane-associated mGlu5 was surprisingly not associated with a decrease in DHPG-stimulated pErk levels. We cannot rule out the presence of mGlu1, which is also activated by DHPG and linked to the pErk pathway. In addition, the pharmacological concept of “spare receptors” comes into mind, where the number of receptors exceeds the number of effectors present which are needed for a particular response. Thus, even after a reduction of receptor number by more than 50%, there will be enough “spare” receptors to activate the pathway with similar efficiency. Activation of the Akt pathway was not seen here, similar to other studies [36].

Brief exposure to type I mGluR agonists or prolonged delivery of low-frequency stimulation onto the Schaffer collateral pathway leads to LTD in hippocampal slices in vitro as well as in vivo [37]. Studies of the Fragile X syndrome showed that the increased mGlu5-cell surface mobility and synaptic clustering of mGlu5R and NMDAR lead to enhancement of both forms of mGlu5-dependent LTD in the hippocampal CA1 [38, 39]. We observed here a reduced targeting of mGlu5 to the PSD in *Gopc* KO mice, which is likely to have consequences for mGlu5-mediated signaling. Supported by previous studies, the altered mGlu5 activation and its downstream signaling might be responsible for the facilitation of DHPG-induced mGlu5-dependent LTD in Gopc-deficient mice. This may be due to an increase in the proportion of extrasynaptic mGlu5 receptors [40]. In general, activation of group I mGluRs leads to dephosphorylation and activation of STEP, which transiently dephosphorylates tyrosine residues in GluA2-containing AMPARs at the PSD [41]. Downstream processing of AMPARs may be facilitated in KO, as it involves lateral diffusion of AMPARs to extrasynaptic endocytic zones, from which internalization occurs. Besides mGlu5 and NMDA receptor, p38 has been suggested as a key modulator of hippocampal mGlu5-dependent LTD [42]. The increased amounts of extrasynaptic mGlu5 receptors may promote internalization of AMPARs via activation of the p38 MAPK signaling pathway, which stimulates the formation of the GDI-Rab5 complex. p38 MAPK signaling was shown to be increased here in the hippocampus of KO mice and may therefore be responsible for increased LTD in KO mice.

Noteworthy, there is an increase in extrasynaptic mGlu5 during synaptic homeostatic plasticity induced by a chronic increase in neuronal activity, which is due to an upregulation of Homer1a expression which anchors mGluR1/5 extrasynaptically in contrast to Homer1b/c, which anchors mGlu5 perisynaptically. Homer1a-supported mGluR1/5 extrasynaptic signaling results in global downregulation of excitatory synaptic inputs via a mechanism shared with that involved in the induction of LTD, culminating in endocytosis of GluA2 receptors [43]. However, in our study, the basal synaptic transmission was unaffected, suggesting that it is unlikely that Gopc deficiency upregulated Homer1a and by this manner increased extrasynaptic mGlu5 expression.

The fact that the function of mGlu5 is most strongly affected in Gopc-deficient mice is consistent with the deficit in the contextual fear conditioning as we observed here. A similar behavioral phenotype was seen in mGlu5-deficient mice, which also exhibited reduced memory in this assay [44, 45]. Thus, it seems possible that the learning deficits in the Gopc-deficient mice may at least partially be caused by the alterations in mGlu5 targeting and mGlu5-dependent synaptic plasticity.

## Supplementary Information

Below is the link to the electronic supplementary material.

Supplementary file1 (DOCX 172 KB)

## Data Availability

All data relating to this manuscript are included in the main text, in the figures, and in the supplemental data. Materials such as recombinant DNA samples are available from the corresponding author upon request.
