# Supplementary material for: The Golgi-Associated PDZ Domain Protein Gopc/PIST Is Required for Synaptic Targeting of mGluR5
Source: Mol Neurobiol. 2021 Aug 12;58(11):5618–34. doi: 10.1007/s12035-021-02504-9 (PMC8599212; doi:10.1007/s12035-021-02504-9)
Supplement: Supplementary file 1 — Supplementary file1 (DOCX 172 KB) [file 12035_2021_2504_MOESM1_ESM.docx]

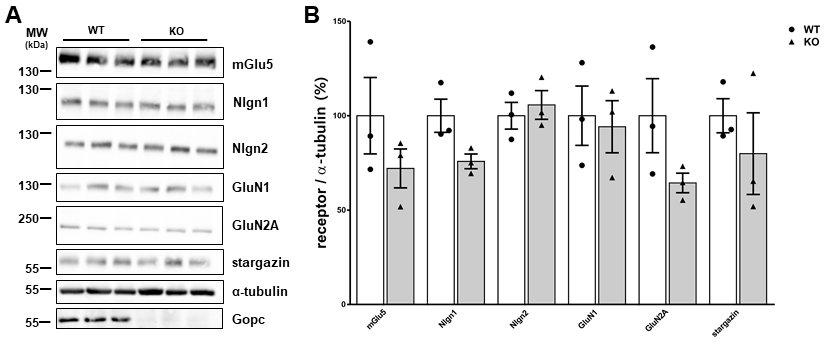


**Supplemental Figure S1. A.** P2 membranes were prepared from whole forebrains from WT and Gopc KO mice. Equal protein amounts were analysed by western blotting using the antibodies indicated. **B.** Data are plotted as the ratio of receptor signal divided by α-tubulin signal (means + SEM, n=3).


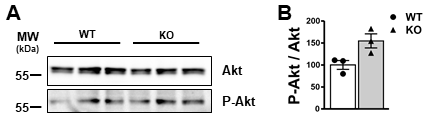


**Supplemental Figure S2. A.** Total forebrain lysate from WT and Gopc KO mice was analyzed by western blotting using antibodies against the phosphorylated and total form of Akt kinase, as indicated. B. Quantitative analysis of the data shown in A. Data are plotted as the ratio of phosphorylated signal divided by signal obtained for total forms of Akt kinase (means + SEM, n=3).
